# Towards an Architecture of a Multi-purpose, User-Extendable Reference Human Brain Atlas

**DOI:** 10.1007/s12021-021-09555-2

**Published:** 2021-11-26

**Authors:** Wieslaw L. Nowinski

**Affiliations:** John Paul II Center for Virtual Anatomy and Surgical Simulation, University of Cardinal Stefan Wyszynski, Woycickiego 1/3, Block 12, room 1220, 01-938 Warsaw, Poland

**Keywords:** Human brain atlas, Brain atlas architecture, Brain atlas definition, Brain atlas platform, Implementation, Neurosurgery, Neuroeducation

## Abstract

Human brain atlas development is predominantly research-oriented and the use of atlases in clinical practice is limited. Here I introduce a new definition of a reference human brain atlas that serves education, research and clinical applications, and is extendable by its user. Subsequently, an architecture of a multi-purpose, user-extendable reference human brain atlas is proposed and its implementation discussed. The human brain atlas is defined as a vehicle to gather, present, use, share, and discover knowledge about the human brain with highly organized content, tools enabling a wide range of its applications, massive and heterogeneous knowledge database, and means for content and knowledge growing by its users. The proposed architecture determines major components of the atlas, their mutual relationships, and functional roles. It contains four functional units, core cerebral models, knowledge database, research and clinical data input and conversion, and toolkit (supporting processing, content extension, atlas individualization, navigation, exploration, and display), all united by a user interface. Each unit is described in terms of its function, component modules and sub-modules, data handling, and implementation aspects. This novel architecture supports brain knowledge gathering, presentation, use, sharing, and discovery and is broadly applicable and useful in student- and educator-oriented neuroeducation for knowledge presentation and communication, research for knowledge acquisition, aggregation and discovery, and clinical applications in decision making support for prevention, diagnosis, treatment, monitoring, and prediction. It establishes a backbone for designing and developing new, multi-purpose and user-extendable brain atlas platforms, serving as a potential standard across labs, hospitals, and medical schools.

## Introduction

An enormous explosion of human brain atlas projects with diverse objectives and spans is witnessed recently (Amunts et al., 2014; Hess et al., 2018; Nowinski, 2021a). These atlases are applicable across research, education and/or clinics, and each of these fields has its own specific purposes, requirements, problems, and challenges.

The wide use of brain atlases in the research focuses mainly on how to integrate and openly share huge amounts of heterogeneous, experimental data in a common reference atlas space and to link these data across scales. Numerous and diverse brain atlases developed by various centers cause problems with standardization. Managing vast amounts of data requires powerful (often leading-edge) and expensive computing systems, which are unaffordable in education and not necessary in atlas-assisted clinical applications.

The brain atlas in education serves students and educators as a visual and interactive aid, enhanced with fully parcellated and labeled virtual brain models with an intuitive and friendly user interface, able to convey the cerebral complexity in a more manageable, rapid, and understandable manner.

Clinically, brain atlases are useful tools for supporting and enhancing diagnosis, treatment, and prediction. They support various domains, mainly neurosurgery, neuroradiology and neurology, with diverse requirements and tools. In particular, in neurosurgery, powerful and dedicated atlas-aided tools are needed for planning, intraoperative support, and postoperative assessment. Extendable brain atlases are also means for aggregation of patient’s specific image and non-image data.

The tremendous brain atlas developments can be considered in terms of atlas concept, content, applications, functionality, and availability. The role and understanding of a human brain atlas have been changing over time along with new acquisition techniques introduced and data acquired, concepts proposed, and applications and tools developed. Not infrequently, the terms like brain image directory, brain inventory, neuroimage repository, brain database, or brain template are used interchangingly with the term “brain atlas”. In the Merriam-Webster dictionary, the term “atlas” (“brain atlas” is missing there) is defined as a) “a bound collection of maps often including illustrations, informative tables, or textual matter” or b) “a bound collection of tables, charts, or plates” (URL, Merriam-Webster/atlas), which captures the essence of the early stereotactic print atlases. Different authors define and/or use brain atlases in various ways. Roland and Zilles (1994) consider brain atlases as a research tool to make generalizations about localization of function and structure at both the macroscopic and microscopic level and to study variations in gross morphology and microstructure. They define conventional brain atlases as collections of micrographs or schematic drawings of brain sections with identified anatomic (invariable) structures from one or a few brains, whereas in computerized atlases the structures are deformable. According to Toga (2002), digital atlases provide semantic and spatial information that can be used to link together the rich collections of data from disparate sources. Moreover, Toga et al. (2006) describe brain atlases for basic and clinical brain research, and establish criteria for an ideal atlas for this purpose. Boline et al. (2008) regard digital brain atlases as useful references and analytical tools, and as a framework for data sharing. Evans et al. (2012) consider brain atlases as large-scale neuroimaging databases that capture the mean and variance in the population. Mandal et al. (2012) discuss population- and disease-specific brain templates as a neuroscientific research tool. According to Amunts et al. (2014), the human brain atlases are central to integrate in a topographically meaningful manner diversified information about numerous aspects of the human brain, such as micro- and macrostructural parcellation, connectivity, regional functional specialization, and temporal dynamics. Kuan et al. (2015) consider the electronic brain atlas as a means for integrating diverse information in a meaningful manner, understanding complex brain anatomy as well as localizing experimental data and planning experiments. Hess et al. (2018) address the usage of brain atlases as a neuroimaging research tool for analysis of brain structure and function. Bjerke et al. (2018) employ reference atlases in *The Human Brain Project* (Amunts et al., 2016) as a means to integrate neuroscience research data from healthy and diseased brains to increase data sharing.

In the above approaches, the brain atlases are perceived as research tools, however, these atlases also have wide application beyond research, including education and clinical practice, as overviewed e.g. in (Nowinski, 2021a). For instance, Mori et al. (2013), to systematically utilize a vast amount of imaging information available in medical record systems, such as PACS (Picture Archiving and Communication System), consider the brain atlas as a potential tool suitable for image structurization through atlas-based image parcellation. Oishi et al. (2019) refer to the brain atlas as a three-dimensional (3D) brain image with a set of reference labels, such as a parcellation map, whose core value is being a teaching file of brain anatomy and function. I have defined earlier an ideal brain atlas devised for stereotactic and functional neurosurgery as a population-based, self-growing, structural–functional multi-atlas system composed of four major units, brain models, knowledge database, tools, and clinical results (Nowinski, 2008). The concept of this atlas emerged from a neurosurgery platform with a probabilistic functional atlas expandable by its users that we developed earlier and made it freely available for use to plan surgery and grow the atlas content by the neurosurgical community (Nowinski et al., 2002a). This platform represented a paradigm shift in human brain atlas development from manufacturer-centric to community-centric.

To capture the state-of-the-art in human brain atlases I have overviewed the evolution of these atlases in four categories, content, applications, functionality, and availability (Nowinski, 2021a). Content-wise, electronic human brain atlases were categorized into eight groups taking into account their scope, parcellation, modality, plurality, scale, ethnicity, abnormality, and a mixture of them (overall, the atlas content developments in all these groups are heading in 23 various directions). An explosion of human brain atlas projects with diverse goals, scopes, and sizes is propelled by brain big projects and initiatives, including *The Allen Brain Atlas* (Sunkin et al., 2013), *The Human Connectome Project* (Van Essen et al., 2013), *The Big Brain* (Amunts et al., 2013), the *Brainnetome* project (Fan et al., 2016; Jiang, 2013), *The CONNECT* project (Assaf et al., 2013), *The BRAIN Initiative* (*Brain Research through Advancing Innovate Neurotechnologies*) (BRAIN Working Group, 2014), *The Blue Brain Project* (Markram et al., 2015), *The Human Brain Project* (Amunts et al., 2016), the *Chinese Color Nest Project* (Zuo et al., 2017), and the *Brain/MINDS (Brain Mapping by Integrating Neurotechnologies for Disease Studies*) project (Sadato et al., 2019). A conclusion from this overview in the evolution of human brain atlases is that the brain big projects keep generating vast, ever-growing amounts of data and result in the development of more advanced and complex brain atlases empowered with more sophisticated tools. These efforts keep increasing an atlas landscape inhomogeneity resulting in the difficulty in the atlas standardization as well as the integration and interpretation of various outcomes. Furthermore, as the majority of these efforts is dedicated to brain atlas-related research, a growing imbalance and chasms among research, clinical, and educational applications of human brain atlases can be anticipated.

To counterbalance this brain atlas inhomogeneity trend, it was recommended in (Nowinski, 2021a) to standardize three components, the brain atlas coordinate system, the core reference cerebral model, and the brain atlas platform architecture. The generalized architecture of the human brain atlas shall embody a multi-purpose platform, supporting research, clinical, and educational applications and enable its users, particularly clinicians but also researchers and educators, to grow the initial core brain atlas content with their own data.

This work addresses the last component, a human brain atlas architecture. Based on my experience in the development of 35 commercial human brain atlases and several working prototypes (licensed to 67 companies and institutions, and distributed in about 100 countries), I extend and generalize the concept of the abovementioned ideal brain atlas to support a wide range of applications with diverse requirements while taking into account the recent progress in human brain atlasing. This generalization is in a form of a novel brain atlas architecture with functional units, modules, and sub-modules, and information flow among them. When designing this architecture, it is assumed that the brain atlas supports education, research, and clinical applications with numerous tools, and that it is dynamically and easily extendable directly by its user with his/her own and also other data. Typically, the process of brain atlas creation and extension is separated from that of the brain atlas use. Brain atlas creation is a complicated, tedious and time-consuming process, however, any updates of a core atlas can be carried out directly by its user if properly supported by the atlas platform. The architecture presented here allows the brain atlas user to partly unite these two processes. It is assumed that the user shall be able to perform content extension and update; atlas individualization for the subject- and patient-specific scans; atlas navigation; and atlas-enabled exploration for knowledge presentation and communication in education, knowledge acquisition and discovery in research, and/or decision making support in clinical applications.

The purpose of this work is three-fold: (i) to introduce a new definition of a reference human brain atlas, (ii) to propose a novel human brain atlas architecture that establishes a backbone for designing and developing new, multi-purpose and user-extendable brain atlas platforms, and (iii) to present how to implement such a brain atlas platform derived from the proposed architecture taking into account our three-decade long human brain atlas developments and a plethora of available resources including methods, tools, atlases, and libraries.

The rest of the paper is organized as follows. First, brain atlas-enabled operations as well as atlas-related tools and functionality are briefly overviewed and systematized. Next, a new definition of the reference human brain atlas is introduced. Then, a novel architecture of a multi-purpose, user-extendable reference human brain atlas is proposed along with a detailed description of its components (i.e., the functional units, modules, and sub-modules), their functionality, and information flow among them along with several scenarios of use. Subsequently, the design and implementation aspects of such a human brain atlas platform are discussed including the design principles and requirements, atlas content, data primitives and structures, object design and management, implementation (of the functional units, modules and sub-modules), functionality, user interface, and software engineering issues including software layered architecture. Moreover, we provide numerous examples of available relevant resources, including algorithms, methods along with their overviews, frameworks, atlases, toolkits, databases, libraries, repositories, and web links, which are useful in this implementation. Finally, this work is discussed and summarized.

## Atlas-Related Functionality

Various tools and operations are necessary for atlas employment ranging from atlas creation to providing functionality supporting a wide spectrum of diverse atlas applications. We distinguish four atlas-related groups of tools and operations, namely, for atlas creation and extension, individualization, navigation, and enabling its use for knowledge acquisition, aggregation, presentation, discovery, communication, and decision making support.

Atlas creation tools are diverse means useful for building new atlases, editing the existing atlases, and extending them with new atlas content and/or modules. These tools can be grouped into stand-alone and atlas built-in. Examples of stand-alone tools include the *Medical Imaging Interaction Toolkit* (MITK) integrating two other popular toolkits, the *Visualization Toolkit* (VTK) (URL, vtk) and the *Insight Toolkit* (ITK) (Wolf et al., 2005; URL, itk); *SPM* for a neuroanatomical variability assessment (Ashburner, 2009; URL, spm); the *Vascular Editor* to create and edit vascular (and generally tubular-like) networks (Marchenko et al., 2010); *FreeSurfer*, a suite of tools for a cortical surface generation (Fischl, 2012; URL, freesurfer); *FSL*, a library of analysis tools for functional, structural and diffusion neuroimages (Jenkinson et al., 2012; URL, fsl); *3D Slicer*, a free open-source software application for medical image computing providing versatile visualizations, automated segmentation, and registration (Fedorov et al., 2012; URL, slicer); and *BioImage Suite* for image visualization, registration, and surface editing (URL, bioimage suite). Our experience indicates that the tools directly integrated with the atlas have an advantage by enabling any new atlas content to be created and edited within the already existing neural context (Nowinski et al., 2012a, b). Hence, to facilitate the user to extend and/or update the existing atlas(es) by him/herself, the built-in atlas tools are preferable.

Atlas individualization is carried out by its registration to patient-specific data. Numerous image registration methods have been developed from conceptually simple and very fast that do not require any parameter setting, such as the Fast Talairach Transformation (Nowinski et al., 2006b) to very sophisticated methods grouped into point-based, surface matching, and whole image content-based methods with numerous similarity measures as reviewed by Ardekani et al. (2005), Klein et al. (2009), Sotiras et al. (2013), Ou et al. (2014), and Viergever et al. (2016).

Atlas navigation encompasses tools for brain atlas content browsing, including content selection and manipulation. The content selection ranges from choosing a suitable reference plate to composing a desirable scene of interest from the entire (multi)atlas content with its possible virtual dissecting for subsequent manipulation, animation, examination, and quantification. Manipulation includes standard operations for rotating, zooming, panning, and view setting.

The atlas-enabled operations allow the user to explore and use the atlas as a tool for knowledge presentation, communication, aggregation, and discovery; atlas-assisted processing and analysis of neuroimages; and atlas-guided decision making support. These operations can be performed on bare or individualized atlas. Largely, two major groups of atlas-enabled operations are distinguished: general and application-specific.

### Atlas-Enabled General Operations

Atlas-enabled general operations are widely applicable and useful across multiple atlas applications. We distinguished two groups of such operations, common (typically employed) and advanced (emerging).

The general common operations include four main functions: segmentation (structure delineation), labeling (structure identification and annotation), querying, and quantification.

A brain atlas to be suitable for neuroimage segmentation and labeling by its individualization shall be deformable, fully parcellated, and completely labeled. For atlas-assisted brain image segmentation, the abovementioned image registration methods can be employed. Moreover multi-atlases, as being more effective than single atlases, have been recently applied for this purpose (Artaechevarria et al., 2009; Lötjönen et al., 2010; Wu et al., 2016).

Labeling varies from a simple identification and naming of the segmented structures to more advanced annotating operations including vessel diameters (Nowinski et al., 2011), vascular variants (Nowinski et al., 2009a), and pathology description (Nowinski et al., 2014b).

Either the cerebral model (or the brain map) or the index can be queried. A simple way of querying the cerebral model is by getting its label at a pointed location. An easy way of querying the index is by its text searching along with highlighting the searched structure in the cerebral model.

Quantification provides various measurements, such as distance, angles, areas, and volumes. It also includes value reading, such as coordinates provided that the atlas is stereotactic and probabilities for probabilistic atlases.

The common operations can also be performed on a bare atlas, such as *VOXEL-MAN* (Hoehne, 2001) or Cerefy (Nowinski & Thirunavuukarasuu, 2004). This type of use is mainly in neuroeducation for self-education and classroom teaching. The brain atlas can also be employed offline clinically for referencing while being placed beside the displayed patient-specific scan. Initially in this way, *The Electronic Clinical Brain Atlas* (Nowinski et al., 1997a) had been used next to a surgical workstation to plan neurosurgery before its content was directly integrated with surgical workstations, such as the *StealthStation* (Nowinski, 2009).

The advanced operations may facilitate handling big, computer-consumable brain atlas data to easier and faster understand them, and to discover knowledge. These data are available in various forms, such as brain image repositories, probabilistic atlases, and multi-atlases. These advanced operations may include complex quantification, comparison and searching, and they may originate from various fields, such as artificial intelligence (AI) as well as Virtual and Augmented Reality (VR/AR). AI and deep learning (Zaharchuk et al., 2018) can be employed to find in neuroimage data cost-effectively the relevant patterns that may be beyond humans’ abilities. VR technology (Anthes et al., 2016) and advanced 3D display technologies (Yang et al., 2016), including holography applied for building holographic brain atlases (Petersen et al., 2019), enhance and facilitate exploration of big and heterogeneous in nature data. It is considered that the use of machine learning and virtual reality has the potential to significantly enhance our understanding of how neuronal activity underlies cognition and behavior (BRAIN Working Group, 2014).

### Atlas-Enabled Application-Specific Operations

Atlas-enabled application-specific operations are customized to specific tasks and they vary in terms of field of application and the way of atlas use. I illustrate some of them here, based on my experience in designing and developing atlas-based applications in five areas, neuroeducation for automatic testing of brain knowledge, brain research in psychiatry to automatically generate the atlas-derived regions and volumes of interest (ROIs/VOIs) for image analysis in population imaging studies, human brain mapping for locus-driven analysis of activation loci, stroke management for automatic atlas-driven quantitative analysis of ROIs/VOIs, and neurosurgery for access planning and evaluation.

To evaluate student’s knowledge in neuroeducation, a testing module was designed and incorporated into *The Cerefy Atlas of Brain Anatomy* (Nowinski et al., 2002b), based on a method presented in (Nowinski et al., 2009b). This module is for both self-testing and classroom testing. The approach exploits a two-way mapping between the image and the index. First, the instructor is able to set the testing parameters, including the scope of tested knowledge, scoring points, and the number of attempts. Then, a random generator selects items from the numbered index (list) while avoiding repetition. To test the location and naming of cerebral structures, two types of queries are presented to a student: “Where is?” and “What is?” When the name of the selected item is highlighted in the index (testing against “Where is?”), the student aims to point to this selected structure in the image. When the selected structure is shown in the image (testing against “What is?”), the student aims to indicate the name of this structure in the index. After a random selection of all the structures, the module provides the total score and the time spent doing testing.

Automatic generation of ROIs or VOIs by means of brain atlases is useful in research in population studies. Then, relevant ROIs/VOIs statistics can be automatically compared between, for instance, the left and right hemispheres and/or patients and healthy controls. This approach was employed to study schizophrenic patients with and without passivity and to compare them with healthy controls by calculating fractional anisotropy in the corresponding atlas-generated VOIs (Sim et al., 2009).

A different approach of the ROIs/VOIs use is to determine them in a brain scan and analyze their content by means of the brain atlas. These ROIs/VOIs can correspond to particular features in the scan, such as lesions. Having the brain atlas individualized, its contribution to each ROI/VOI is then automatically quantified. This contribution includes the list of atlas structures overlapping with the ROIs/VOIs, and a contributing volume and percentage of each structure. The approach was used in ischemic stroke image processing to support decision making in thrombolysis by automatically and quantitatively analyzing MRI images and perfusion maps (Nowinski et al., 2006a). Then, the infarct and penumbra regions were taken as the VOIs and quantified by atlases of neuroanatomy and blood supply territories.

A locus-driven analysis of functional images in human brain mapping is a variation of this approach (Nowinski & Thirunavuukarasuu, 2003). Then, ROIs are taken as the activation loci in functional images. The atlas of anatomy extended with Brodmann’s areas was employed for automatic labeling of the loci with the names of structures and their stereotactic coordinates. These loci were marked on the images with the superimposed atlas, and their list was provided (Nowinski et al., 2000b).

In neurosurgery, the atlas facilitates to plan an access corridor to the target structure. The atlas determines all the structures encountered along the planned corridor and around it, enabling the neurosurgeon to assess various potential corridors in the process of decision making. Particularly in stereotactic and functional neurosurgery, the atlas can be used pre-, intra-, and post-operatively (Nowinski, 2001). Pre-operatively, the atlas enables target and trajectory planning, and provides the list of trajectory-intersected structures. The use of multiple atlases increases both the quality of planning and the surgeon’s confidence (Nowinski et al., 2000a, 2010). Intra-operatively, the atlas provides the actual structure where the tip of the electrode is located, the structures already intersected by the electrode, distances to critical structures, and the anatomic and vascular surrounding context (Nowinski et al., 2010). Post-operatively, the atlas facilitates to analyze the correctness of placement of the deep brain stimulating electrode or a permanent lesion.

## Definition of the Reference Human Brain Atlas

Numerous definitions of a brain atlas have been proposed by various authors as overviewed in the Introduction section. The majority of these definitions are research-centric. Here I introduce a new definition of the reference human brain atlas supporting our main requirements, namely, that the atlas shall be multi-purpose and user-extendable, meaning that the atlas shall serve education, research, and clinical applications, as well as that the user shall be able to extend the atlas with new content, including his/her own clinical data.

To meet these requirements, I define the atlas as follows: *the reference human brain atlas is a vehicle to gather, present, use, share, and discover knowledge about the human brain with a highly organized content, tools enabling a wide range of its applications, massive and heterogeneous knowledge database, and means for content and knowledge updating and growing by its user*.

This definition is constructive and it shall guide the design of the architecture of the multi-purpose, user-extendable reference human brain atlas.

## Human Brain Atlas Architecture

**Fig. 1:**
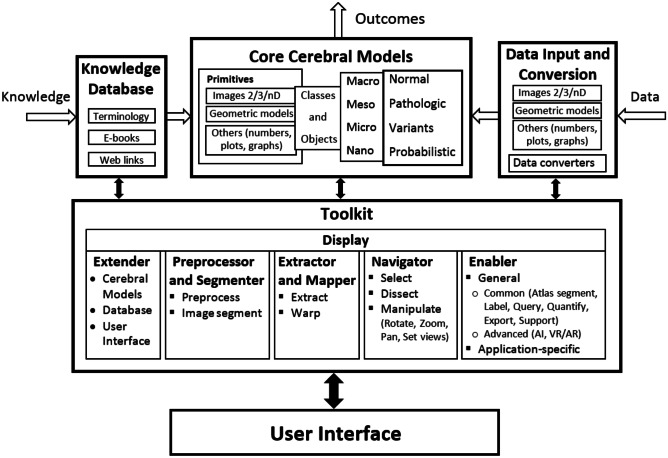
The architecture of the multi-purpose, user-extendable reference human brain atlas with its major components, encompassing the functional units, modules (with sub-modules), connections, data primitives and structures, and functionality. The flow of information is illustrated by arrows. The open arrows indicate the input of data and knowledge, and the output of outcomes; these arrows are unidirectional. The filled (black) arrows indicate control among the functional units and the user interface, and they are bi-directional

The term “architecture” is defined in the Merriam-Webster dictionary as “the manner in which the components of a computer or computer system are organized and integrated” (URL, Merriam-Webster/architecture). The brain atlas architecture shall encompass all major components of the atlas in use, namely, the atlas content, atlas-related tools, and atlas handled data. The proposed architecture of the multi-purpose, user-extendable reference human brain atlas is presented in Fig. 1. This architecture is composed of four functional units, each with multiple modules (often subdivided into sub-modules), all united by the user interface. The units are *Core Cerebral Models*, *Knowledge Database*, *Data Input and Conversion*, and *Toolkit.* The modules within each unit are mutually connected.

Below I enclose a detailed description of the functional units, their modules, and sub-modules.

### Core Cerebral Models

The *Core Cerebral Models* (*CCMs*) represent the content of the brain atlas. They are characterized by data primitives with type and dimensionality forming objects and classes within the scale and scope.

There are three types of atlas data primitives: images, geometric models, and others. Images are n-dimensional arrays, including 2D, 3D, and nD. Examples of 2D images are tomographic images or color-coded maps, 3D images are volumetric images, and nD images are multi-modal images or time-dependent volumetric images (like time series or volumetric images of development, aging, or disease progression). The image representation also includes textures (such as employed in *The Human Brain, Head and Neck in 2953 Pieces* atlas (Nowinski, 2017b) to provide dissections and a triplanar within a 3D geometric scene).

Geometrical models may result from the segmentation of anatomical structures (Nowinski, 2017b) or pathology lesions from images, like ischemic infarcts (Nowinski et al., 2006a) or hemorrhages (Nowinski et al., 2014c), or they can be synthesized, like 3D vascular variants (Nowinski et al., 2009a). Geometrical models can take the form of 2D contours like polylines or splines (since when superimposed on the scans they do not obscure the image content (Nowinski et al., 2006a)), 3D curves (e.g., to represent white matter nerve fibers (Nowinski et al., 2012a)), or 3D polygonal surfaces (Nowinski, 2017b).

Under others, there are multiple, diverse data, such as numbers (parameters, coordinates), plots, and graphs. Examples of numbers in brain atlasing include an incidence rate of anatomic variants (Nowinski et al., 2009a), location (coordinates) and geometry (size) of deep brain stimulating electrodes (Nowinski et al., 2003), or values of patient-specific parameters such as stroke scales (Nowinski et al., 2014a). A plot is a 1D function that may represent an electrical signal or a spectrum in magnetic resonance spectroscopy. A graph can be a means to represent brain connections (Arsiwalla et al., 2015), as neuronal networks, generally, are high-dimensional graphs packed into 3D tissue at enormously high density (Helmstaedter, 2013).

The scale of the *CCMs* encompasses atlas data at gross (macro), meso, micro, and nano levels.

The scope covers normal brain content, pathologic content capturing neurologic diseases, models of variants, and distributions (maps) of probabilities. In this architecture, the population-based content is categorized by two distinct groups: variants and probabilistic maps. Probabilistic maps represent statistics of the whole population (obtained usually by averaging) capturing mainly its mean and standard deviation, whereas variants include all the instances in the population capturing the individual variation (for instance, this is a suitable way of presenting the arterial variants of an incomplete circle of Willis as averaging all these variants does not make sense).

### Knowledge Database

The *Knowledge Database* (*KD*) represents, in general, the annotations of the *CCMs*. This vast, heterogeneous, and ever-growing knowledge pool is grouped into three modules with various (not necessarily non-overlapping) content: *Terminology*, *e-Books*, and *Web links.*

The *Terminology* module provides terminologies, indices, nomenclatures, and ontologies that shall be complete, consistent, and systematic.

The *e-Books* module contains descriptions of cerebral structures, systems, and disorders, mainly in a form of electronically published material and/or printed textbooks available in a digital format, such as PDF. The employment of e-book materials facilitates both atlas design and development as well as its use.

The *Web links* module comprises other links to a plethora of web brain-related resources, encompassing databases, repositories, tools, and publication libraries.

### Data Input and Conversion

The *Data Input and Conversion* (*DIC*) unit fulfills two main roles: loads input research and/or clinical data and provides data conversion. The input data can be used for atlas-assisted analysis, decision making support, and/or continuous updates of the *CCMs*. *CCMs* updating is accomplished by providing functions for adding, removing, replacing, and/or editing the *CCMs* content. These updates have the same format of data type as that of the *CCMs*. Examples of brain atlas content update include new best stereotactic targets (Nowinski et al., 2003), geometric models of cerebrovascular variants (Nowinski et al., 2009a), neurologic parameters (Nowinski et al., 2014a), and any new neuroimages and/or scans to be aggregated within the population or pathologic scopes.

The *DIC* contains two major modules, *Loader* and *Converter*. *Loader* provides functions to load data in any format supported by the *CCMs*. *Loader* shall be able to read a wide spectrum of file formats, including image file formats to input neuroimages, 3D file formats to handle 3D models, and XML (to handle, for instance, graphs representing brain connections (Arsiwalla et al., 2015) and the Scalable Vector Graphics (SVG) image format employed in some brain atlases (Bakker et al., 2015; Majka et al., 2013)). This module also provides data anonymization.

*Converter* provides functions for conversion of numerous image file formats as well as conversion from image to non-image data and vice versa which is required for data integration. For instance, brain connection data are available in various representations, such as a volumetric representation (Maier-Hein et al., 2017), geometric representation (as 3D curves for individual fibers and transparent polygonal models for complete white mater tracts) (Nowinski et al., 2012a), or graphs (Arsiwalla et al., 2015), and the integration of these diverse datasets requires prior conversion. In particular, the module shall also handle the conversion of contours to binary images; multiple contours to color-coded maps; and polygonal models to volumetric images and vice versa, as polygonal models are easier for handling and scene composing, whereas volumetric images are more suitable for volume editing at a voxel level (e.g., for simulation of access corridors by voxel removal in neurosurgical training and planning).

Both input data and outcomes may require conversion; the latter is also needed to enable 3D printing by converting, for instance, 3D polygonal cerebral models in the OBJ format to STL or VRML formats.

### Toolkit

The *Toolkit* unit represents the functionality that supports brain knowledge gathering, presentation, use, sharing, and discovery. It is divided into six modules corresponding to major groups of supporting functions: *Extender*, *Preprocessor and Segmenter*, *Extractor and Mapper*, *Navigator*, *Enabler*, and *Display.*

#### Extender

The role of *Extender* is to link the *DIC* with the *CCMs* and *KD* to enable dynamic extension or updating of the atlas content by its user. *Extender* supports three main scenarios: 1) *CCMs* only are extended or updated; 2) *CCMs* and *KD* are extended or updated, or 3) *CCMs*, *KD,* and the user interface are extended or updated.

The *CCMs* can be updated completely or adaptively. The complete update requires the re-calculation of the entire atlas (regeneration of the whole model) as opposed to the adaptive update that increments the existing atlas with the updated data. These two ways of atlas update are illustrated in the probabilistic stroke atlas (Nowinski et al., 2014a) that are employed depending on a selected method of data aggregation. Adding a new object (a structure or lesion) will require to update, besides the *CCMs*, also the *KD* with the updated index. Extending the scope, scale, and/or class requires updating the *CCMs*, *KD,* and the user interface (for instance by including additional panels). This update is easily achievable by employing the *CCMs* object representation and management as discussed below.

#### Preprocessor and Segmenter

The *Preprocessor and Segmenter* module comprises two sub-modules, *Preprocessor* for image preprocessing and *Image-based Segmenter* for image-guided segmentation of brain images in health and disease.

Neuroimage preprocessing, such as artifact removal, image enhancement, and/or intensity non-uniformity (bias) correction, may be required prior to atlas-assisted analysis, which is particularly important in the processing of brain pathologic images as reviewed by Cenek et al. (2018).

Brain segmentation is a key step in image processing in neuroscience, diagnosis, and treatment planning as well as in brain atlas development. Numerous methods have been proposed for brain image segmentation as reviewed by Cabezas et al. (2011), Despotović et al. (2015), González-Villà et al. (2016), Helms (2016), Wu et al., (2016), Dora et al. (2017), Mirzaei and Adeli (2018), including the state of the art reviews on pathologic brain segmentation (Mortazavi et al., 2012; Gordillo et al. 2013; Menze et al., 2015; Wadhwa et al., 2019; Ghaffari et al., 2020).

#### Extractor and Mapper

The *Extractor and Mapper* module contains two sub-modules, *Extractor* to provide functions for feature extraction from a scan, and *Mapper* for providing functions for atlas or data warping.

The extraction of some specific features may be required for subsequent image warping in the landmark-based registration methods and/or for atlas-assisted ROI/VOI analysis, including the study of symmetric patterns between the brain’s hemispheres. Examples of specific features extracted by dedicated algorithms include point landmarks, distributed landmarks, structures, and lesions. Some features, such as the cortical surface, may be obtained by employing *Segmenter*.

*Mapper* (or atlas individualizer) enables the atlas to be overlaid on the loaded patient-specific data (in brain scan interpretation) or the scan on the atlas (in human brain mapping). Numerous methods are suitable for image warping as reviewed and compared by Ardekani et al. (2005), Klein et al. (2009), Sotiras et al. (2013), Ou et al. (2014), Viergever et al. (2016).

#### Navigator

*Navigator* provides means for arranging suitable atlas content for its subsequent exploration. It contains sub-modules to select (*Selector*), dissect (*Dissector*), and manipulate (*Manipulator*) atlas content.

*Selector* enables content selection at several levels, scope, scale, class, object, and variant, including any specific scene to be assembled/disassembled by the user from a composable atlas content.

*Dissector* dissects virtually volumetric data and polygonal models by means of 3D cutting in predefined and arbitrary planes or surfaces to expose structures lying inside.

*Manipulator* provides functions for real-time manipulation of the composed scene including rotation (arbitrarily or around any reference axis), zooming, scrolling, and panning; as well as setting views (for the predefined views or any user-specified position and/or orientation). This manipulation is performed in a smooth and continuous manner enabling the user for an uninterrupted tracking of any object(s) of interest.

#### Enabler

*Enabler* provides functions for atlas-enabled, -guided, and/or -supported operations. As overviewed above, these operations are divided into two main groups: general and application-specific. The general operations, in turn, are divided into common and advanced.

The common general operations are embedded into six sub-modules, *Atlas-based Segmenter, Labeler, Querier, Quantifier*, *Exporter,* and *Supporter*.

*Atlas-based Segmenter* handles the results of atlas-guided segmentation generated by applying *Mapper*. The segmented content can be managed as color-coded regions, contours, polygonal objects, volumetric objects, or time series for nD data.

*Labeler* sets the presentation of the annotated information and adaptively clears it. Atlas-generated labeling is automatic. Moreover, free text annotations are available enabling the user to add to the automatically generated label any supplementary text which is not available in the index. In addition, label saving and editing are provided for capturing and enhancing the labels placed on the annotated image or scene.

Label clearing can affect all the placed and displayed labels, only the last one (e.g., to correct it), or any user-selected label to facilitate nice placement of numerous labels.

*Querier* enables structure querying by probing either the atlas content (*CCMs*) or the atlas index (*KD*) in terms of structure naming and localization (i.e., “What is?” or “Where is?”). When querying the content, the name of the queried object along with its complete annotation (i.e., the label) is provided as defined by the *KD* and set in *Labeler*. When querying the index, the queried object is identified and indicated within the *CCMs* content.

This sub-module also enables a more advanced and wider scope of probing concerning diverse features, including geometry (e.g., provide all the vessels with a diameter lower than a given value), proximity (e.g., show all the neighboring structures within a given distance), and topology (e.g., provide all the structures supplied by a selected blood vessel or show all the structures innervated by a selected white matter tract or nerve).

*Quantifier* provides functions for various measurements and quantification. It measures distances (along straight and curved lines), angles, areas, and volumes for any selected atlas content (like an individual structure or a tissue class). It provides various statistics (like means, standard deviations, moments, and similarity measures) in user-defined regions and volumes of interest as well as the variability in the whole population (or any subset of the specimens) including a temporal variability in the modeling of disease progression or brain development. This sub-module also enables a continuous atlas reading at a rolled-over location by providing coordinates for stereotactic atlases and probabilities for probabilistic atlases.

*Exporter* exports a selected content of the *CCMs* along with the annotations both from the *KD* and these placed by the user. This sub-module performs three main functions for the user-composed and annotated scene 1) captures its image and saves it to an external file; 2) exports this scene (such as all component geometric objects) to an external file; and 3) creates for this scene or any selected structure a file for 3D printing. In this way, the user (mostly the educator) is able to generate teaching and/or presentation digital materials as well as solid models.

*Supporter* provides miscellaneous supporting functions, including parameter setting, getting information, and providing help.

*Advanced operations*. This group of operations, especially advantageous to handle big data to discover knowledge from them, requires advanced modules, like AI to extract knowledge from the *CCMs* by identifying immensely complex patterns along with their context; VR to extend and expedite exploration capabilities of *Navigator*; high-end modelers and simulators at all levels of brain organization; and metadata management and advanced analysis, quantification and comparison, for instance, to quantify the shapes of structures in shape modeling analysis and to compare various brain maps.

*Application-specific Operations* shall support a wide range of diverse applications for neuroscience research, neuroeducation, neurosurgery, neurology, neuroradiology, and psychiatry, among others. Working examples of such operations have been discussed above.

#### Display

*Display* visualizes data for any of the *CCMs* formats and supports all five toolkit modules. The image display provides a wide range of functions from displaying single images to grid images to multi-planar reformatted images to maximum and minimum intensity projections to triplanar to surface, volume and hybrid rendering for navigation and exploration to visualization of time series of volumetric data and polygonal models for nD images.

### Information Flow

From an information flow standpoint, the presented architecture may be considered as a system with an input and output. The input changes the output and/or the internal state of the system as a result of employing *Extender*. The information flow is shown in Fig. 1 by arrows. The open arrows indicate the input of data and knowledge, and the output of outcomes; these arrows are unidirectional. The filled (black) arrows indicate control among the functional units and the user interface, and they are bi-directional.

The user interface controls all six modules of *Toolkit*. *Toolkit* in turn is bi-directionally connected to the other three units, the *CCMs*, *KD*, and *DIC*. The *CCMs* and *KD* have their local editable databases, while the remaining functional units are for processing and communication. The *CCMs* can be updated externally via the *KD* and *DIC* and internally by means of *Extender*. *Toolkit* contains six modules, internally bi-directionally connected, five for processing and manipulation and one (*Display*) for presentation. Three *Toolkit* modules work typically as a pipeline from atlas individualization (by *Extractor and Mapper*) to atlas browsing (by *Navigator*) to quantitative exploration (by *Enabler*).

Several possible scenarios of use and the resulting information flow among the functional units and modules can be envisaged. An educator employs, for instance, *Navigator* to browse the *CCMs* to select relevant 2D images while viewing them with *Display*, and then *Enabler* to export these images to her own application. A self-training student uses *Navigator* to select, manipulate, and dissect 3D objects, and then *Enabler* to label, query, and quantify them while continuously presenting the effects by *Display*. A neuroscientist imports his research data via the *DIC*, registers a relevant part of the *CCMs* with the data through *Extractor and Mapper*, and uses *Navigator* and *Enabler* to manipulate and explore the annotated and quantified data while checking all the results with *Display*. A neurosurgeon imports a patient’s scan through the *DIC*, generates an individualized atlas by employing *Extractor and Mapper*, selects relevant slices via *Navigator*, performs their labeling and quantification by utilizing general functions of *Enabler*, plans surgery through application-specific operations of *Enabler*, and finally exports the surgery plan via *Exporter* while monitoring all the outcomes with *Display*. An atlas manager updates the content by importing relevant material via the *DIC* and integrating it with the *CCMs* by means of *Extender*. A neuroradiologist imports a patient’s scan via the *DIC*, preprocesses it and segments focal lesions (i.e., determines VOIs) by employing *Preprocessor and Segmenter*, generates an individualized atlas using *Extractor and Mapper*, performs an atlas-based VOI analysis through *Application-specific Operations* provided by *Enabler*, and checks the outcomes using *Display*. Additionally, the neuroradiologist queries the pathologic content of the *CCMs* by employing *Enabler.* Similar cases present there can provide decision support; if none is available, the interpreted case can be added to the pathologic *CCMs* by means of *Extender*.

## Brain Atlas Platform Design and Implementation

The proposed architecture with its detailed description is constructive and serves as a guideline for developing multi-purpose and user-extendable brain atlas platforms. However, it is still high-level and provides some flexibility in designing such a brain atlas platform, resulting in potential various implementation instances. Moreover, this is the most complex architecture of all the brain atlases and atlas-based applications we have developed so far. Therefore below, we illustrate how such a brain atlas platform could be implemented based on our experience and existing resources. We discuss here design principles and requirements, content, data structures, functionality, implementation of functional units with modules and sub-modules, user interface, and software engineering issues.

### Design Principles and Requirements

The three main atlas design principles are to be multi-purpose, user-extendable, and reference. The content-wise requirements include nD, multi-scale, multi-scope, fully parcellated, completely labeled, stereotactic, detailed, accurate, realistic, of high resolution, spatially consistent, extendable (scalable), composable, dissectible, explorable, and modular. Moreover, the brain atlas platform shall have a rich set of functionality, support a wide spectrum of image and data formats, and be interactive, user friendly and affordable.

### Content

Some initial 2D image content of the *CCMs* can be imported from the *NOWinBRAIN* repository containing over 5,700 images of the human brain, head, and neck publicly available at (URL, nowinbrain). An example of 3D content is *The Human Brain, Head and Neck in 2953 Pieces* (Nowinski et al., 2015; Nowinski, 2017b), a 3D holistic atlas created based on a paradigm “brain from blocks”, which is freely available at (URL, 2953 atlas). Although this atlas already comprises about 3,000 components, the model requires further extension (e.g., to add more systems and the spinal nerves, and to extend the spinal cord) and subdivision of some structures (such as the thalamus) into finer components. How to grow the already created hierarchical content of this atlas both upwards and downwards is proposed in (Nowinski, 2017a). An alternative approach to content creation is its import from the numerous existing brain atlas resources as reviewed in (Nowinski, 2021a) such as, for instance, the *Brainnetome Atlas* project investigating the hierarchy in the human brain from genetics to neuronal circuits to behaviors (Jiang, 2013) including spatiotemporal changes during development or aging (Fan et al., 2016).

### Data Structures

The proposed architecture determines data primitives as images (2D, 3D, and generally nD), geometric models (2D and 3D), and others (numbers, plots, and graphs). They are grouped into objects and classes, and further with respect to scale (i.e., macro, meso, micro, and nano) and scope (normal, pathologic, variants, and probabilistic).

To facilitate navigation these data shall be organized, for instance, into albums (as in the *NOWinBRAIN* gallery (Nowinski, 2021b; URL, nowinbrain) or modules (as in *The Human Brain, Head and Neck in 2953 Pieces* atlas (Nowinski, 2017b)). Moreover to facilitate querying, these data structures require further organization and extension with derived information, such as volumes of structures, physical properties of structures, and lengths and diameters of vascular segments as well as cranial and spatial nerves. Furthermore to enable advanced querying, certain intelligent and semantic data structures are desirable containing, for instance, anatomic-functional relationships (as in *The Cerefy Atlas of Brain Anatomy* (Nowinski et al., 2002b)), normal-pathology relationships (as in the *3D Atlas of Neurologic Disorders* (Nowinski et al., 2014b)), relevant descriptions and references (as in *The Cerefy Atlas of Cerebral Vasculature* (Nowinski et al., 2009c)), as well as hierarchical (topological) relationships as in *3D Slicer* by employing the *Medical Reality Markup Language* (Fedorov et al., 2012) or *BrainInfo* (URL, braininfo) providing for each structure its superstructures and substructures.

Examples of intelligent and/or semantic data representations were proposed, for instance, by Hoehne et al. (1995) and Mechouche et al. (2009). Hoehne et al. (1995) presented a new representation of knowledge concerning human anatomy and function using an intelligent volume approach. The knowledge representation consists of a semantic network linked to a digital volume representation by employing relations such as “Part Of”, “Projecting To”, “Supplied By” and “Is A”. Mechouche et al. (2009) used symbolic knowledge described in a formal ontology represented in the Ontology Web Language to facilitate knowledge sharing. An ontology introduces a shared vocabulary describing various aspects of the modeled domain and provides a specification of the meaning of this vocabulary (Horrocks, 2008). Then to label cortical structures, the mereo-topological relations between the various cortical structures were employed.

### Implementation of Functional Units, Modules, and Sub-Modules

The implementation of the functional units, modules, and sub-modules can benefit from the implementations of our brain atlases developed so far, in particular, the latest and most advanced *The Human Brain, Head and Neck in 2953 Pieces* (Nowinski et al., 2015).

#### Implementation of Core Cerebral Models Unit

The 2D images of the *CCMs*, which are predominantly for neuroeducation, can be organized, for instance, into brain image collections, similarly to *NOWinBRAIN* - a systematic, comprehensive, extendable, and web-based repository with over 5,700 images (Nowinski, 2021b; URL, nowinbrain). *NOWinBRAIN* has been implemented by employing an open-source image library software *Piwigo* (URL, piwigo). Piwigo organizes the images into hierarchically arranged albums and sub-albums, and provides functions for image display, resizing, scrolling, searching, saving, and downloading by its user. Then, the 2D images of the *CCMs* can be arranged into albums corresponding to the scope, each further subdivided into sub-albums in terms of the scale, classes, and image content.

The 3D cerebral models of the *CCMs* can be implemented as geometric or volumetric. The advantages of geometric objects over volumetric representation are high (sub-voxel) resolution and parcellation, easy extendibility, easy editability, and feasibility of structure-to-structure conflict detection, whereby avoiding structure intersection resulting in unique labeling. Object representation and management within a single atlas can benefit from that implemented in *The Human Brain, Head and Neck in 2953 Pieces* atlas (Nowinski, 2017b). All the (polygonal) structures there are divided into virtual segments, including tissue class cluster (brain, head, neck, and systems), tissue class, group within a tissue class, and individual component within a tissue class (with or without its sub-components), with additional classification into the left side, right side, and middle. To uniquely define a brain atlas component, a pair of identifiers (IDs) is required (tissue class ID, structure ID), so the atlas objects are represented as a grid of tissue classes (modules) and each module as a list of structures. A tissue class cluster is realized as a set is tissue class IDs and a tissue class group as a band of structure IDs. An individual structure is annotated with a vector of features (including its name) and assigned its appearance in terms of color and transparency. This atlas distinguishes 17 tissue classes, while, for instance, *NOWinBRAIN* (version 1.1) introduces 26 tissue classes further subdivided into 207 subclasses as building blocks (Nowinski, 2021b; URL, nowinbrain). This representation is advantageous, allowing the user to easily edit and extend the content as well as to quickly compose any 3D scene for exploration from the components, like from the Lego blocks, supporting the paradigm “from blocks to brain”.

For the implementation of the *CCMs* the above approach has to be extended from a pair to a quintuple of IDs, namely, (scope ID, scale ID, class ID, object ID, variant ID). The scope ID determines a normal, pathologic, variants', or probabilistic scope. The scale ID determines a macro, meso, micro, or nano scale. The class ID determines a tissue class for the normal, variants', and probabilistic scopes, and a pathologic class for the pathologic scope. The object ID determines a structure for the normal, variants', and probabilistic scopes, and a lesion for the pathologic scope. A variant ID determines a variant for the variants’ scope and is void for the other scopes. An example of pathologic class implementation is in the *3D Atlas of Neurologic Disorders* providing regional anatomy disorders, cranial nerve disorders, and vascular disorders along with a simulation in each pathologic class various lesions annotated with signs, symptoms and syndromes cum description from neurologic textbooks (Nowinski et al., 2014b). An example of a large database of pathological images, including neuropathology, with 20,000 patient cases and 200,000 images is *STATdx* (URL, statdx).

For operations that require the voxel representation, such as image warping, a geometric model can be converted into a volumetric model by voxelization.

#### Implementation of Knowledge Database Unit

The *KD* unit comprises three modules, *Terminology*, *e-Books*, and *Web links*. To implement the *Terminology* module, multiple resources can be utilized. Several print atlases comprise original indices, such as Schaltenbrand and Wahren (1977), and Talairach and Tournoux (1988) that are embedded in electronic atlases, like *The Cerefy Clinical Brain Atlas* (Nowinski & Thirunavuukarasuu, 2004). The most popular terminology, available both in English and Latin, is *Terminologia Anatomica* (Federative Committee on Anatomical Terminology, 1988). We have employed it in *The Human Brain, Head and Neck in 2953 Pieces* atlas (Nowinski et al., 2015). A recent revision of *Terminologia Anatomica*, covering the central nervous system, peripheral nervous system, and sensory organs, is *Terminologia Neuroanatomica* (TNA, 2017).

The implementation of the *e-Books* module can follow realizations in some of our brain atlases that are annotated with e-book materials in various ways. For instance, *The Cerefy Atlas of Brain Anatomy* (Nowinski et al., 2002b) is provided with the functional description of structures in a form of labels. *The Cerefy Atlas of Cerebral Vasculature* (Nowinski et al., 2009c) is enhanced with the description of vascular anatomy, variability, and references. The *3D Atlas of Neurologic Disorders* (Nowinski et al., 2014b) annotates disorders along with the corresponding signs, symptoms, and syndromes as automated labels and additionally provides a disorder description from neurology textbooks available in the PDF format. The content of the *e-Books* module may also be in a form of web-links to publisher’s databases of images, such as Elsevier’s *STATdx* (URL, statdx), and e-books, such as *Thieme MedOne Neurosurgery* (URL, Thieme MedOne Neurosurgery) or *Thieme MedOne Radiology* (URL, Thieme MedOne Radiology).

For the implementation of the *Web links* module, neuroscience resources can be exploited, such as the *Neuroscience Information Framework* (URL, neuinfo) or *BrainInfo* (URL, braininfo). The *Neuroscience Information Framework* maintains the largest searchable collection of neuroscience data (with about 2,000 databases), accessible tools, atlases, and the largest ontology for neuroscience on the web. *BrainInfo* is a portal to neuroanatomical information with 15,000 neuroanatomical terms along with hierarchical relations of each structure to its superstructures and substructures. This resource may also be useful in enhancing any teaching materials that are derived from the atlas. Similarly *NOWinBRAIN*, a systematic and freely available repository of more than 5,700 3D reconstructed anatomic images, which is useful in preparing neuroanatomic courses (Nowinski, 2021b; URL, nowinbrain).

#### Implementation of Data Input and Conversion Unit

The *Loader* module of the *DIC* handles various image file formats that are employed in medical imaging (Graham et al., 2005; Larobina & Murino, 2014). The most frequently used is DICOM (Digital Imaging and Communication in Medicine) (URL, DICOM) and other popular formats include ANALYZE™ (Robb et al., 1989) and MINC (Medical Imaging Network Common Data Format) (URL, minc). Clinical imaging data are usually stored and transferred in the DICOM format, however, it is complicated, manufacturer-dependent, and different modalities may require special processing during conversion (Li et al., 2016). Therefore, the neuroimaging community has widely adopted a much simpler format NIfTi (Neuroimaging Informatics Technology Initiative) and NIfTI-1 which is adapted from the widely used ANALYZE™ 7.5 file format (URL, nifti).

To implement *Loader*, there are several free DICOM image viewing and processing software packages (Escott & Rubinstein, 2003); moreover, a list of the top 25 best free DICOM viewers, such as *PostDICOM*, *Horos*, *RadiAnt*, *ProSurgical 3D*, *MicroDicom*, and *DICOM Web Viewer*, is provided at (URL, DICOM viewers). For instance, *PostDICOM*, considered as one of the best viewers, is compatible with Windows, Mac OS X, and Linux, can be operated from android and iOS-based devices, and provides cloud-based PACS services (URL, postdicom). There is also a plethora of commercial DICOM viewers, such as *OsiriX* (URL, osirix). Several DICOM viewers provide additional, more advanced, features, including, image manipulation, image fusion, making measurements, and patient anonymization (URL, DICOM viewers).

For the implementation of the *Converter* module of the *DIC*, several available freeware packages for image conversion can be employed, including *XnConvert*, *CoolUtils Online Image Converter*, *FileZigZag*, *Zamzar*, *Adapter*, *Free Image Convert and Resize*, *PixConverter*, *SendTo-Convert*, and *BatchPhoto Espresso* (URL, image converters). For instance, *XnConvert* (*XnView*), considered the most advanced, provides 80 operations and is able to read from more than 500 input formats (including DICOM) and write to around 80 output file formats (URL, xnview). Moreover, *XnConvert* is multi-platform available for Windows, OS Mac, and Linux, and multi-lingual with more than 20 translations. Furthermore, some DICOM viewers also provide image conversion, such as *RadiAnt*, *MANGO*, and *Sante DICOMdir Viewer Pro* (URL, DICOM viewers). The conversion from DICOM to NIfTI as the first step in the analysis of neuroimaging data is addressed in (Li et al., 2016).

For 3D file conversion, several software tools are available, including *Meshconvert*, *Spin 3D*, *Online CAD Converter*, *i3DConverter*, and *Greentoken* (URL, 3D file converters). For instance, *Meshconvert* is free and supports 37 input formats, including OBJ and STL (URL, meshconvert), whereas *i3Dconverter* is commercial and handles 815 file formats (URL, i3dconverter). Alternatively, an open-source *Open Asset Import Library* can be used as a general-purpose 3D model converter (URL, asset library).

#### Implementation of Toolkit Unit

To implement the *Extender* module of *Toolkit*, there are several resources for handling 2D images and 3D models. The implementation of 2D image update is straightforward by employing software packages, such as the above-discussed *Piwigo* (URL, piwigo).

To implement 3D model updating in the *CCMs*, four approaches can be distinguished for model creation, namely, external general or dedicated, and internal general or dedicated. Externally created models have to be imported via the *DIC*. Internally, models are created by *Extender*.

There are several general (external) 3D modelers, such as *Blender*, a free and open-source tool for 3D modeling and painting (URL, blender); *TinkerCAD*, a constructive solid geometry free software enabling to build complex shapes from simple primitive shapes (URL, tinkercad); *FreeCAD*, an open-source 3D parametric modeler (URL, freecad); *SelfCAD*, a web-based 3D modeling application, free for educational use (URL, selfcad); *Rhinoceros 3D*, a commercial free-form 3D modeling and editing tool that handles polygonal meshes and NURBS (Non-Uniform Rational Based-Splines) curves (URL, rhino3d); *ZBrush*, a digital sculpturing commercial software that combines modeling, texturing and painting for creating high-resolution models (URL, zbrush); and *3Ds Max*, commercial software for polygonal and NURBS modeling, and painting (URL, 3ds max).

To build our 3D brain atlases, we have developed one external general free-form mesh modeler (Nowinski et al., 2012b), and two external dedicated modelers, a tubular modeler for modeling blood vessels and cranial nerves (Marchenko et al., 2010), and a tract modeler for modeling white matter tracts (Nowinski et al., 2012a). Our internal general 3D modeler (Nowinski et al., 2012b) enables editing of the imported models to fit them to the existing neural content, paint them to enable color-derived labeling, and to a limited extent create new models.

To enable implementation of the *Preprocessor and Segmenter* module of *Toolkit*, several open-source tools are available for neuroimage processing, including frameworks, such as *Freesurfer* (Fischl, 2012; URL, freesurfer), *3D slicer* (Fedorov et al., 2012; URL, slicer), *BrainSuite* (Shattuck & Leahy, 2002), *ITK-snap* (Yushkevich et al., 2006), and *ImageJ* (Schneider et al., 2012) as well as libraries, such as *SPM* (Statistical Parametric Mapping) (Ashburner, 2009; URL, spm), *FSL* (Jenkinson et al., 2012; URL, fsl), *ANTS* (Advanced Normalization Tools) for multidimensional image segmentation, registration and statistics (URL, ants), and *ITK* (Insight Segmentation and Registration Toolkit), an object-oriented cross-platform extensive library for image processing, segmentation, and registration (URL, itk). For automated brain tumor segmentation, *BraTS Toolkit* providing deep learning-based algorithms can be employed (Kofler et al., 2020).

The *Extractor and Mapper* module of *Toolkit* has two sub-modules, *Extractor* and *Mapper*. *Extractor* can be implemented based on dedicated algorithms for extraction of specific structures and/or regions, for instance, the anterior and posterior commissures (Bhanu Prakash et al., 2006), interhemispheric fissure (Hu & Nowinski, 2003), ventricular system (Xia et al., 2004), and white and gray matter (Gupta et al., 2010). General features, such as cortical surface, are extracted by *Image-based Segmenter.*

The implementation of *Mapper* is enabled by employing open-source tools, such as *ITK* with leading-edge registration algorithms (URL, itk) and *ANTS* providing various transformations and similarity metrics (URL, ants).

#### Implementation of Navigator Module

The implementation of the *Navigator* module of *Toolkit* may follow smooth and continuous navigation realized in *The Human Brain, Head and Neck in 2953 Pieces* atlas (Nowinski et al., 2015; Nowinski, 2017b).

#### Implementation of Enabler Module

The *Enabler* module of *Toolkit* comprises seven sub-modules, *Atlas-based Segmenter, Labeler, Querier, Quantifier*, *Exporter, Supporter*, and *Application-specific Operations*.

*Atlas-based Segmenter* handles the results of atlas-guided segmentation and its implementation can benefit from that in a multi-atlas (Nowinski et al., 1997b) managing 11 brain atlas volumes with diverse representations.

*Labeler* handles automatic and interactive annotations and its implementation can follow diverse implementation approaches taken in our brain atlases. In particular, atlas-generated labeling is automatic and characterized by type, dimensionality, appearance, and plurality. The label type can be transient (shown temporarily at the rolled-over location), mouse movement independent (placed permanently on the image), and scene manipulation independent (remaining on the manipulated 3D scene until cleared). The label dimensionality can be 2D, 3D, or generally nD. The label can automatically appear as 1) a settable text at a pointed location with a user-determined font, size, and color; 2) a settable text at the end of a line segment of a variable-length with a settable size, type, and color whose distance and end are determined interactively by the user; or 3) as a settable text, settable line segment, and an arrowhead with a settable size and type pointing to the labeled structure. Label plurality can be in terms of 1) atlases (a single label of a multi-atlas is shown at a time versus a simultaneous display of labels generated by all the atlases (like in Nowinski et al., 2006a); and 2) features, i.e., a single feature (e.g., structure name) or multiple features, like a vessel name and its diameter at a pointed location (Nowinski, 2017b) or a structure name along with a disorder cum description resulting from structure’s damage (Nowinski & Chua, 2013).

When implementing *Querier*, two-way content-index querying can follow that in our family of the *Brain Atlases in 1492/1969/2953 Pieces* (Nowinski & Chua, 2014; Nowinski et al., 2011, 2015). Advanced querying requires suitable data models. To query vascular geometric relationships, tubular modeling with the centerline and diameter at any location can be employed to model the arterial (Volkau et al., 2005) and venous systems (Volkau et al., 2008). To query topological relationships among structures, hierarchical relationships, such as presented in (Fedorov et al., 2012; URL, braininfo), and intelligent models for medical knowledge representation, such as proposed by (Hoehne et al., 1995; Mechouche et al., 2009), are required.

The implementation of *Exporter* can benefit from that of *The Human Brain, Head and Neck in 2953 Pieces* atlas (Nowinski et al., 2015) by extending it from images to other data types.

The implementation of *Supporter* can follow that of *The Human Brain, Head and Neck in 2953 Pieces* atlas (Nowinski et al., 2015).

The implementation of *Application-specific Operations* can follow those in our brain atlases as discussed above in the section on *Atlas-enabled application-specific operations*.

#### Implementation of Display Module

The implementation of the *Display* module of *Toolkit* is enabled by employing the *Visualization Toolkit VTK*, an open-source, freely available software system for 3D computer graphics, modeling, volume rendering, and scientific visualization, that supports a broad variety of visualization algorithms and advanced modeling techniques (URL, vtk). There are also several brain network visualization tools, such as *BrainNet Viewer* and *Connectome Viewer*, as overviewed by Xia et al. (2013). Moreover, some DICOM viewers, such as *postDICOM*, *Horos*, *RadiAnt*, *Navegatium*, *Pro Surgical 3D*, *MicroDicom*, *3DimViewer*, *Athena DICOM Viewer*, and *Miele LXIV*, provide advanced image display, including multiplanar reconstruction, maximum and minimum intensity projections, and volume rendering (URL, DICOM viewers).

### Functionality

The description of the functionality for each functional unit, module, and sub-module of the presented human brain atlas architecture has been embedded above in the detailed description of this architecture.

### User Interface

The user interface provides full access to all the available functionality. It shall be user friendly, intuitive with seamless accessibility, and provide smooth and continuous navigation in and exploration of the cerebral space. To implement the user interface, it can be adapted, for instance, from the user interface of the *Allen Brain Atlas*, an integrated portal for exploring the central nervous system, whose developers have continued to provide standardization of the user interface (Sunkin et al., 2013). Another simple and generic solution is a web-based user interface of the *Scalable Brain Atlas* providing instant access to public brain atlases and related content (Bakker et al., 2015). Other examples include the graphical user interface of *3D Slicer* supporting rich functionality (Fedorov et al., 2012) and the *Brainnetome Atlas* viewer with multiple views (Fan et al., 2016).

**Fig. 2:**
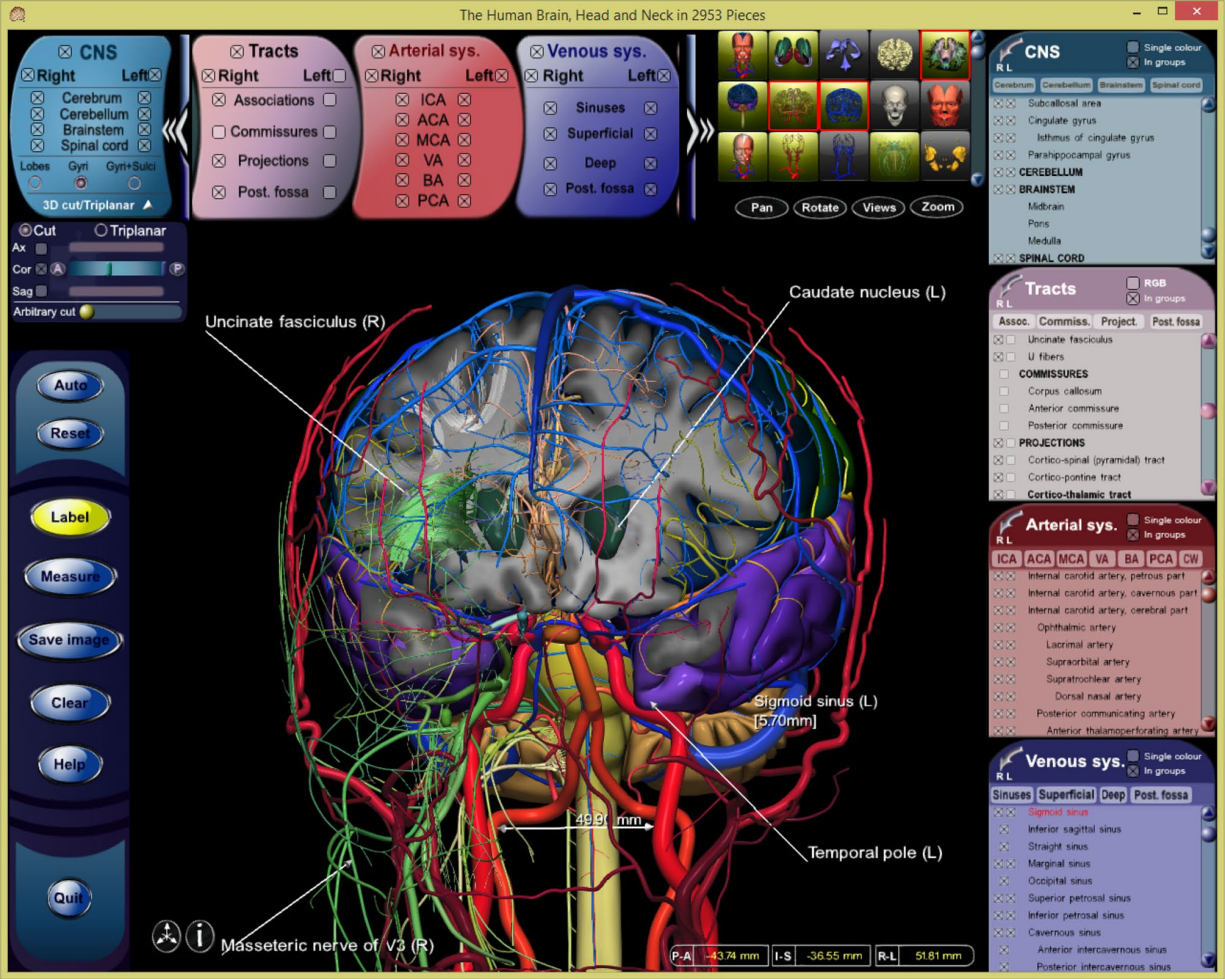
Illustration of the brain atlas user interface along with its selected functionality (from *The Human Brain, Head and Neck in 2953 Pieces* atlas, Nowinski et al., 2015). Components of the user interface: main view with a composed 3D model (center); matrix of selectable tissue clusters and individual tissue classes (top-right) and a manipulation panel below it (additionally, all manipulation functions are mapped into the mouse buttons); horizontally scrollable selected tissue class panels for left/right side and group selections (top-left); indices of all selected tissue classes, each with the list of selectable structures, synchronized with their corresponding panels (right); controls including the dissection and triplanar panel control (left); and info “i” (bottom-left in the main view). Illustration of selected functionality: atlas content selection (gross anatomy with the cerebrum, cerebellum, brainstem, and cervical spinal cord; deep nuclei; white matter tracts on the right; intracranial arterial system; intracranial venous system; extracranial arterial system; and cranial nerves on the right), virtual brain cutting (anteriorly in coronal direction), permanent (manipulation independent) labeling (with a resizable arrowhead, line segment, and text), quantification (the distance measured between the left internal carotid artery, cervical part and the right vertebral artery, extracranial part), and at the actually pointed structure (the left sigmoid sinus) its transient labeling (with name and diameter), stereotactic coordinates (bottom-left in the main view), and highlighting in the intracranial venous system index (in red)

A solution worth considering is the use and extension of some of the user interfaces in our human brain atlas products and prototypes that we have continually kept improving for over a few decades. Especially, the recent family of our *Brain Atlases in 1492/1969/2953 Pieces* (Nowinski & Chua, 2014; Nowinski et al., 2011, 2015) has a refined, friendly, and intuitive user interface, as illustrated in Fig. 2. Its main features are (1) a single window avoiding multiple, mutually obscuring windows, (2) smooth and continuous navigation in the cerebral space, (3) synchronized panels and indices displayed on-demand, (4) content-focused manipulation (with all main manipulation functions mapped also into the mouse buttons), (5) two-way mapping between the index and the 3D scene, and (6) dynamically composed (or decomposed) and explorable 3D scenes. The key components of this user interface are the main view, tissue matrix, panels with the selectable tissue classes, indices of all the selected tissue classes (each with the list of selectable structures) synchronized with their corresponding panels, and controls. As this user interface corresponds to a single atlas, it has to be extended accordingly to the new content and functionality. Namely, the main view remains as is to show the results of *Display* and an axillary panel is needed to present the content of the *KD*; the tissue matrix is substituted with a multi-level grid to enable selection of scope, scale, and classes; the mutually synchronized class panels and indices remain as are; and the controls must be substantially extended to include the complete functionality of *Toolkit*. This proposed user interface may be further enhanced with new developments in 3D displays and interaction as discussed in (Nowinski, 2017a).

### Software Engineering Issues

Some software engineering issues arising in implementing our brain atlas platforms we addressed earlier, e.g., in (Nowinski et al., 1997b, 2002a, 2012b), however, the proposed architecture is more complex than those of any of our previous atlases.

The first generation of our brain atlases which we started in the 1990s (Nowinski, 2017a) was implemented in Macromedia Director with a Lingo scripting language. This popular multimedia authoring platform (now discontinued) enabled the creation of multi-platform solutions. However, with the increasing complexity of the atlas content and the use of 3D models this authoring platform was not able to ensure real-time interaction, despite the continual increase in the computer processing power. Consequently, the family of the *Brain Atlases in 1492/1969/2953 Pieces* (Nowinski & Chua, 2014; Nowinski et al., 2011, 2015) was developed in C++ with the Open Graphics Library (OpenGL), the industry standard for high performance graphics (URL, opengl). This brain atlas family comprises 5 atlases and despite a substantial increase in the atlas content in consecutive atlases, their interactivity was sustained. Therefore to implement the proposed brain atlas architecture, it is recommended to use C++ with OpenGL. Alternatively, game engines can be exploited, such as Unreal Engine (URL, unrealengine), Unity (URL, unity), and CryEngine (URL, cryengine). A list of 12 free game engines is given at (URL, gameengines), whereas (Christopoulou & Xinogalos, 2017) provide an overview and comparative analysis of them.

Then, the software architecture of the discussed brain atlas platform can be envisaged as having four layers: 1) base layer with the operating system, file system, C++ , and hardware drivers; 2) library layers with OpenGL and/or game engines; 3) resource layer with the above presented toolkits, libraries, links and other relevant resources; and 4) core layer corresponding to the proposed brain atlas architecture.

## Discussion

This works stems from three roots: (a) a conclusion and recommendation from a comprehensive overview on the evolution of human brain atlases (Nowinski, 2021a) to establish the standardized architecture of a human brain atlas; (b) it follows my previous work on the architecture of an ideal stereotactic brain atlas (Nowinski, 2008) and that of a 3D interactive and reference brain atlas (Nowinski et al., 2012b) as well as on the probabilistic functional atlas (Nowinski et al., 2003) and the probabilistic stroke atlas (Nowinski et al., 2014a) designed to be extendable by an individual user or even the whole community; and (c) it shares my long-term experience in the development of 35 brain atlas products with a continual refined content, functionality and user interface, as well as numerous research, education and clinical brain atlas prototypes as overviewed in (Nowinski, 2017a). To my best knowledge, this is the first paper on the architecture of a multi-purpose, user-extendable reference human brain atlas.

The designed architecture is novel as corroborated by the below state-of-the-art review. PubMed under „human brain atlas architecture” gives 150 results (as of 26 May 2021). However, the majority of these publications are on brain’s architecture (such as network architecture, fiber architecture, connectional architecture, molecular architecture, cytoarchitecture, myeloarchitecture, topological architecture, microvascular architecture, system architecture, cortical architecture, spatiotemporal architecture, functional architecture, microanatomical architectures, anatomical architecture, micro-architecture, structural architecture, circuit architecture, axonal architecture, nuclear architecture, vascular architecture, and architecture of cognition). There are only five references, all weakly relevant, by Brinkley et al. (1997), Gustafson et al. (2004), Liakos et al. (2004), Feng et al. (2005), and Mechouche et al. (2009). Brinkley et al. (1997) described a software framework for developing information systems in anatomy within a distributed architecture that includes spatial and symbolic anatomy information resources, Web and custom servers, and authoring and end-user client programs. Gustafson et al. (2004) presented a software design, implementation and storage architecture of the software that allows the user to rapidly display slices of the digital atlas at any arbitrary slicing angle. Liakos et al. (2^004^) proposed a dynamic layered architecture, based on the mediator approach, for the design of a transparent and scalable distributed system that can process large 3D images distributed across several image servers. Feng et al. (2005) presented a Java atlas-viewer for browsing biomedical 3D images and atlases allowing arbitrary re-sectioning of the data and interactive browsing through the volume. The viewer was implemented to run on any workstation using the architecture neutral Java programming language. Mechouche et al. (2009) described an interactive system for the semantic annotation of magnetic resonance neuroimages implemented on a client–server architecture using Web services. Under “software architecture AND human brain atlas” on PubMed, there are 12 citations. The relevant include the already listed five references (Brinkley et al., 1997; Gustafson et al., 2004; Liakos et al., 2004; Feng et al., 2005; Mechouche et al., 2009) and our abovementioned work on the architecture of a 3D interactive and reference brain atlas (Nowinski et al., 2012b).

There are certain, partly relevant, works concerning architectures of animal (particularly rodent) brain atlas-assisted systems for research applications and architectures of non-brain atlas systems. For instance, Lee et al. (2010) presented the *MouseBIRN Atlasing Toolkit* (*MBAT*), a scalable informatics system for unifying and accelerating digital atlasing workflows with a tiered, plug-in architecture to provide a modular and extensible design. The system handles 2D images and 3D volumes. The architecture comprises four workspaces, search workspace, registration workspace, comparison viewer workspace, and hierarchy editor workspace. The system is developed using the Java programming language for cross-platform development with the Java Plugin Framework to manage the plug-ins. The *MBAT* enables researchers to customize all platform components to speedily achieve personalized workflows. Hawrylycz et al. (2011) described the *Digital Atlasing Project* established by the International Neuroinformatics Coordinating Facility (INCF) (URL, incf). This is an international effort to set digital atlasing standards and to design and create an atlas-based data sharing framework for the rodent brain, because constructing an open and shared digital atlasing framework has the potential to transform collaborative research. Majka et al. (2013) developed a 3D brain atlas reconstruction service with a repository of 12 animal brain atlases publically available on the internet in the SVG-based Common Atlas Format. The atlases are available as polygonal meshes, volumetric files and bitmap images, and each atlas contains metadata. The software architecture of this web service contains three layers, presentation layer, server layer, and computation layer. Fedorov et al. (2012) described the architecture of *3D Slicer*, a platform for biomedical engineers, developers, and applied scientists for fast prototyping and efficient development of biomedical image analysis tools. This platform has a layered and modular architecture. At the lower level, there are platform-specific components with the OpenGL graphics library and hardware drivers. Then, there are languages (C++ , Python, JavaScript) and libraries that provide higher-level functionality (including the *Visualization Toolkit* supporting computer graphics and visualization (URL, vtk), the *Insight Toolkit* providing image segmentation and registration (URL, itk), and the *Common Toolkit*, a biomedical image computing library (URL, ctk). At the top, there is *3D Slicer* itself with extensions consisting of the core (the main application framework) and modules (plugins). The core comprises the graphical user interface, logic (controller), and the *Medical Reality Markup Language* which defines the hierarchies of the data elements. The modules are divided into core modules and loadable modules.

Summarizing, this overview confirms that despite several brain atlas architecture-related efforts none of them is aligned with the objectives and requirements of the presented work. On the other hand, there is a plethora of relevant resources, including algorithms, methods, atlases, frameworks, toolkits, libraries, databases, repositories, and web links, which have been considered here when addressing the implementation of a brain atlas platform based on the proposed architecture.

This architecture poses several advantageous features. It supports brain knowledge gathering, presentation, use, sharing, and discovery. Moreover, it is broadly applicable for education, research, and clinical applications. Therefore, we consider it a multi-purpose architecture, meaning an architecture of the human brain atlas as a potentially useful standard not only for the research community but also for other various communities. The importance of standardization of tools and a user interface as well as data integration across time, species, and projects have been addressed, for instance, in the development of the *Allen Brain Atlas*, at least for research applications (Sunkin et al., 2013).

A brain atlas's importance and potential in clinical applications have been appreciated and addressed by a few authors (BRAIN Working Group, 2014; Mori et al., 2013). In fact, the development of atlas-based clinical applications for prediction, diagnosis, and treatment has been a major direction of our research and development work and we share this experience here. Neurosurgery planning and assessment (Nowinski, 2001, 2009; Nowinski et al., 2010) was our first clinical application with anatomic, probabilistic, functional, and vascular atlases created. Our atlas-based solutions in stereotactic and functional neurosurgery have been accepted by the majority of surgical companies and integrated with main surgical workstations (Nowinski, 2009). In addition, we have developed working prototypes in other fields for atlas-assisted stroke management (Nowinski, 2020; Nowinski et al., 2006a), segmentation and labeling of pathological neuroimages with tumors causing a mass effect in brain cancer (Nowinski & Belov, 2005), quantification of cerebral lesions (Nowinski et al., 2006a), and stroke outcome prediction (Nowinski et al., 2014a). A vast, still unexploited, potential of brain atlases in neuroradiology has been addressed in (Nowinski, 2016) describing nine promising applications for which the working prototypes (proofs of concept) we developed earlier.

We also have focused on neuroeducation applications, attempting to go beyond a typical atlas use in brain atlas products, such as *VOXEL-MAN* (Hoehne, 2001) or *Interactive Head & Neck* (Berkovitz et al., 2003). Our novel educational employment of the brain atlas is for self-testing and classroom assessment (Nowinski et al., 2009c) available on computers and mobile devices, interdisciplinary education across neuroanatomy-neuroradiology-neurology (Nowinski & Chua, 2013), generation of teaching materials (Nowinski et al., 2011, 2015), advanced education for residents and clinicians (Nowinski et al., 2014b, 2015; Kockro et al., 2021), and patients’ education and instruction (Nowinski, 2016).

The introduced architecture has been designed from a user’s perspective. It allows the content of the atlas to be extendable with the user’s own and/or other data. In this way, the atlas operator plays a double role both of the atlas user and atlas creator (which roles are typically separated), provided that some core atlas is already available. The user is able to export any part of the atlas content to prepare publications, teaching materials, printed solid 3D models, or import it to some other application. No assumptions have been made regarding the size of content and type of computer platform, so the designed architecture may be considered content-scalable and platform-independent.

The reference human brain atlas is defined here as a vehicle with a preferable online web-based access; common standardized data primitives and formats; efficient data organization, conversion, comparison, aggregation, and sharing; comprehensive description of the brain; and computational methods for automated and accurate data processing and analysis as well as interactive manipulation and visualization. The atlas content shall be multi-scale and multi-scope, organized in a stereotactic space, fully parcellated, completely labeled, and potentially color-coded. The suites of tools and services described and/or referred to shall enable brain knowledge use, accumulation, combination, sharing, and presentation as well as support a wide range of atlas applications in (i) research to discover general brain mechanisms and greatly advance our understanding of how the brain works along with its complex relationships between structure, function, and connectivity in health and disease at various levels; (ii) student- and educator-oriented neuroeducation to convey the cerebral complexity in a more manageable, rapid, and understandable form; and (iii) clinical practice for atlas-assisted prevention, diagnosis, treatment, monitoring, and prediction.

This work has several limitations. Although it proposes a novel human brain atlas architecture, a resultant brain atlas platform has not yet been developed. To ease this limitation, I discuss the brain atlas platform design and implementation, including the design principles and requirements, atlas content, data primitives and structures, object design and management, implementation of the functional units with modules and sub-modules, functionality, user interface, information flow including several scenarios of use by diverse users, and software engineering issues along with software layered architecture. In addition, I provide numerous examples of suitable resources, including algorithms, methods and their overviews, atlases, frameworks, toolkits, repositories, databases, libraries, and web links, that are invaluable in this implementation.

The proposed architecture is oriented to a general user (including clinicians and educators) and it may not fully meet the designer and/or developer’s perspective. Consequently, it differentiates from other, research-oriented platforms, such as, for instance, *MBAT* within *BIRN* (Lee et al., 2010) that focuses on building an infrastructure for collaborative environments or *3D Slicer* supporting biomedical engineers, developers, and applied scientists in fast prototyping and efficient development of image analysis tools (Fedorov et al., 2012). Generally, this is difficult to embody all diverse perspectives, if possible at all, taking into account the breadth of the field and a plethora of existing atlases, available methods, and tools developed by numerous working groups. The designed data structures and the proposed file format converters may not be sufficient and they may require extensions, because of the heterogeneous nature and a very wide range of the countless computational, behavioral, physiologic, anatomic, connectomic, and genetic datasets. Finally, due to unprecedented and unpredictable progress in human brain research and atlasing enabled by technological advances, it is rather hard, if possible at all, to define a stable and long-lasting human brain atlas architecture.

## Summary

Based on my three-decade long experience in brain atlas products and prototype development, a definition of a multi-purpose, user-extendable reference human brain atlas is introduced and the architecture of such an atlas platform is proposed along with its implementation aspects. The reference human brain atlas is defined here as a vehicle to gather, present, use, share, and discover knowledge about the human brain with a highly organized content, suitable tools enabling its wide range of applications, heterogeneous and vast knowledge database, and means for its content and knowledge updating and growing by the users. The architecture contains four functional units, core cerebral models, knowledge database, data import and conversion, and toolkit, all united by a user interface. The data and control flow among the functional units is presented and each unit is described in terms of its component modules and sub-modules, enabling functions, handled data structures, and implementation aspects.

I believe that this architecture is broadly applicable and useful for education, research, and clinical applications in numerous domains. It is designed from a user’s perspective, uniting processes of atlas use and atlas extension by the user, which processes are typically separated. The architecture also supports brain knowledge gathering, presentation, use, sharing, and discovery serving as a potential common standard across labs, hospitals, and medical schools.

## Information Sharing Statement

This architecture proposal is freely sharable. No data nor application is involved to be shared.
